# The TRAR gene classifier to predict response to neoadjuvant therapy in HER2‐positive and ER‐positive breast cancer patients: an explorative analysis from the NeoSphere trial

**DOI:** 10.1002/1878-0261.13141

**Published:** 2021-12-17

**Authors:** Tiziana Triulzi, Giampaolo Bianchini, Serena Di Cosimo, Tadeusz Pienkowski, Young‐Hyuck Im, Giulia Valeria Bianchi, Barbara Galbardi, Matteo Dugo, Loris De Cecco, Ling‐Ming Tseng, Mei‐Ching Liu, Begoña Bermejo, Vladimir Semiglazov, Giulia Viale, Juan de la Haba‐Rodriguez, Do‐Youn Oh, Brigitte Poirier, Pinuccia Valagussa, Luca Gianni, Elda Tagliabue

**Affiliations:** ^1^ Department of Research Fondazione IRCCS Istituto Nazionale dei Tumori Milan Italy; ^2^ Department of Medical Oncology IRCCS Ospedale San Raffaele Milan Italy; ^3^ DRAST Fondazione IRCCS Istituto Nazionale dei Tumori Milan Italy; ^4^ Oncology and Breast Diseases Department Postgraduate Medical Education Center Warsaw Poland; ^5^ Department of Medicine Samsung Medical Center Seoul Republic of Korea; ^6^ Medical Oncology Fondazione IRCCS Istituto Nazionale dei Tumori Milan Italy; ^7^ Taipei‐Veterans General Hospital National Yang‐Ming University Taiwan; ^8^ Koo Foundation Sun Yat‐Sen Cancer Center Taipei Taiwan; ^9^ Hospital Clínico Universitario INCLIVA Biomedical Research Institute Valencia Spain; ^10^ NN Petrov Research Institute of Oncology St Petersburg Russia; ^11^ Hospital Reina Sofia Córdoba Spain; ^12^ Division of Medical Oncology Seoul National University Hospital Cancer Research Institute Seoul National University College of Medicine Republic of Korea; ^13^ Centre des Maladies du sein Hôpital du Saint‐Sacrement CHU de Québec Canada; ^14^ Fondazione Michelangelo Milan Italy; ^15^ Fondazione Gianni Bonadonna Milan Italy

**Keywords:** breast cancer, gene expression profile, HER2, pertuzumab, predictive biomarker, trastuzumab

## Abstract

As most erb‐b2 receptor tyrosine kinase 2 (HER2)‐positive breast cancer (BC) patients currently receive dual HER2‐targeting added to neoadjuvant chemotherapy, improved methods for identifying individual response, and assisting postsurgical salvage therapy, are needed. Herein, we evaluated the 41‐gene classifier trastuzumab advantage risk model (TRAR) as a predictive marker for patients enrolled in the NeoSphere trial. TRAR scores were computed from RNA of 350 pre‐ and 166 post‐treatment tumor specimens. Overall, TRAR score was significantly associated with pathological complete response (pCR) rate independently of other predictive clinico‐pathological variables. Separate analyses according to estrogen receptor (ER) status showed a significant association between TRAR score and pCR in ER‐positive specimens but not in ER‐negative counterparts. Among ER‐positive BC patients not achieving a pCR, those with TRAR‐low scores in surgical specimens showed a trend for lower distant event‐free survival. In conclusion, in HER2‐positive/ER‐positive BC, TRAR is an independent predictor of pCR and represents a promising tool to select patients responsive to anti‐HER2‐based neoadjuvant therapy and to assist treatment escalation and de‐escalation strategies in this setting.

## Graphical abstract

TRAR‐low score is an independent predictor of pathological complete response (pCR) to anti‐HER2 agents in patients with HER2‐positive and ER‐positive breast cancer of the NeoSphere study. The higher risk of relapse observed in these patients likely depends on modification by treatment of tumor proliferation and ER activity. TRAR is a promising tool to assist escalation and de‐escalation of anti‐HER2‐based treatment strategies.

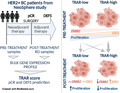

**Abbreviations**
BC: breast cancer
CI: confidence interval
DEFS: distant event‐free survival
ER: estrogen receptor
GEP: gene expression profile
H: trastuzumab
HR: hazard ratio
OR: odds ratio
P: pertuzumab
pCR: pathological complete response
T: taxane
TRAR: trastuzumab advantage risk model

## 1 Introduction

The combination of trastuzumab (H), pertuzumab (P), and chemotherapy has become the standard of care for patients with HER2 overexpressing and/or amplified, that is, HER2‐positive, breast cancer (BC) in the neoadjuvant setting. In the phase II randomized study NeoSphere, a 12‐week long neoadjuvant course with docetaxel (T), H and P significantly increased the rate of pathological complete response (pCR) in the breast as compared with TH, TP, or HP (45.8% versus 29.0%, 24.0%, 16.8%) [1], which led to FDA approval of dual antibody‐based HER2 blockade and chemotherapy in this patient population [2]. Since pCR correlates with long‐term outcome [3], increasing the proportion of patients who respond could have long‐term benefits. The early identification of patients who are unlikely to respond offers the potential to amend neoadjuvant treatment to obtain improved responses. On the other hand, undoubtedly highly effective antibody‐based HER2 blockade may be unnecessary in patients who already benefit from a single anti‐HER2 agent.

In addition to treatment, several biological features are implicated in response to HER2 targeting, including tumor intrinsic subtype, hormone receptor status, alterations in signaling pathways including PI3K, and host factors such as immune response [4, 5, 6]. Yet the recommendation regarding HER2‐targeted agents and chemotherapy in the (neo)adjuvant setting takes into account just HER2 status.

The development of biomarkers for tailoring HER2‐targeted therapy cannot ignore that HER2 targeting agents play their activity not only through the inhibition of HER pathway but also by their inherent antibody characteristics, which affect immune response [7, 8, 9, 10]. At Fondazione IRCCS Istituto Nazionale dei Tumori—Milano (INT), we developed the 41‐gene classifier TRAR, which is able to identify HER2‐positive BC patients with differential risk of relapse upon treatment with adjuvant H and provides reliable predictive information over established clinical factors in the neoadjuvant setting [11, 12]. The discriminatory capability of TRAR stands on its unique feature of including both genes related to HER2 and estrogen receptor (ER) signaling, (*ERBB2*, *C17orf37*, *GRB7*, *ESR1*), and to split tumors according to their immune infiltration and proliferation characteristics [11]. Here, we aimed to assess whether TRAR is associated with pCR and prognosis to single agent or dual antibody‐based HER2 blockade within the NeoSphere trial.

## 2 Materials and methods

### 2.1 Patients and samples

Details on the NeoSphere study (ClinicalTrials.gov number NCT00545688), and its results have been published elsewhere [1]. NeoSphere was a multicenter randomized phase II study in which patients with HER2‐positive BC were stratified by operable, locally advanced, and inflammatory disease and according to hormone receptor status and randomized to preoperative THP, TH, PH, or TP for 12 weeks. After surgery, all patients continued treatment with four cycles of AC followed by H to complete one year of treatment. The primary endpoint was pCR, that is, absence of invasive tumor cells in the breast [1]. The secondary endpoints were 5‐year progression‐free survival and disease‐free survival [3]. The study was conducted in accordance with the Declaration of Helsinki. Approval for the protocol was obtained from independent ethics committees. Written informed consent was obtained from all patients at study entry, which also covered future biomarker research.

### 2.2 Gene expression profile

RNA was extracted from baseline (pre‐treatment) formalin‐fixed paraffin‐embedded (FFPE) core biopsies and surgical FFPE specimens of patients with residual disease (post‐treatment), and gene expression profiling (GEP) was carried out with Affymetrix U133 Plus 2.0 gene chips as previously described [13]. Thirteen patients were not assessable for pCR. The 41‐gene classifier TRAR was computed as the sum + 5.856708 of the weighted logarithmic expression of 36 out of the 41 genes (Table S1) [11]. Proliferation metagene was computed as described [14].

### 2.3 Statistical analysis

The association of TRAR scores, measured on a continuous scale, with pCR as well as with other categorical clinico‐pathological variables, was evaluated by using the nonparametric Wilcoxon test. The strength of the association of TRAR scores with continuous variables was assessed by the Pearson correlation coefficient (*r*). Univariate logistic regression analysis, modeling pCR probability, was implemented for each variable of interest to estimate the odds ratio (OR) and its 95% confidence interval (CI). The predictive performance of TRAR with respect to pCR was further evaluated by resorting to a multivariate logistic regression model by taking into account treatment arm (TH arm as reference) and the available clinico‐pathological variables, that is, age (continuous), ER status (positive versus negative), and tumor type (operable as reference). TRAR was dichotomized according to the cutoff value identified by median value and association with other variables was evaluate by chi‐square test. Survival curves were estimated by the Kaplan–Meier method. The prognostic performance of TRAR with respect to distant event‐free survival (DEFS), which considers only distant relapse ignoring locoregional relapse from the surgery as event, was implemented estimating the hazard ratio (HR) and its 95% CI. All statistical analyses were carried out with r software (http://www.r‐project.org) by adopting a significance level of ≤ 0.05.

## 3 Results

### 3.1 Study cohorts

**Fig. 1:**
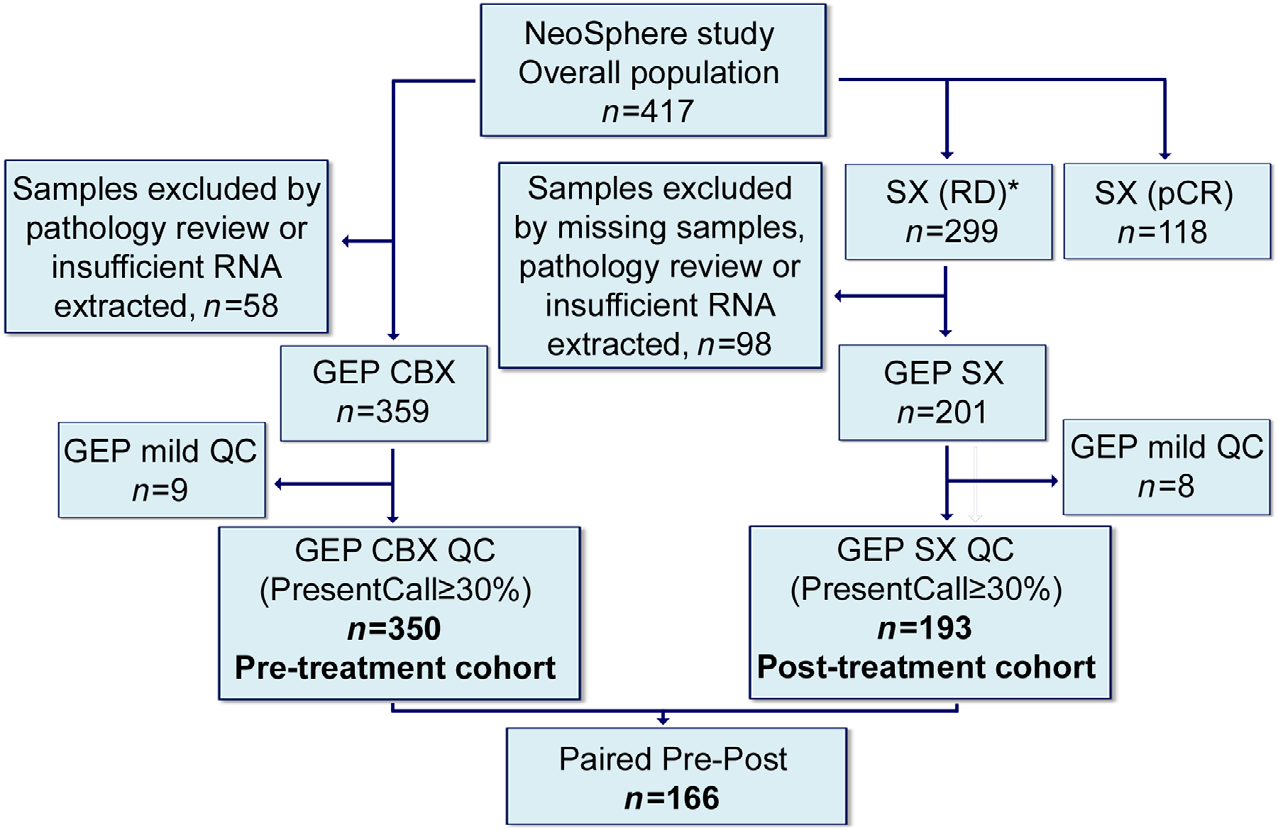
Consort diagram of patients and samples included in the analysis. GEP, gene expression profile; QC, quality check; CBX, core biopsies; SX, surgical samples; RD, residual disease; pCR, pathological complete response. *, includes unknown.

A total of 417 patients were enrolled in the NeoSphere trial, for 350 of them (84%) GEP data were obtained before treatment, as described [13]. Eighty‐seven patients (25%) were enrolled in the TH arm, 92 (26%) in the THP arm, 90 (26%) in the HP arm, and 81 (23%) in the TP arm. No significant differences in terms of baseline patient characteristics and treatment response were observed between the entire NeoSphere and the profiled patient population (pre‐treatment cohort) [13]. Of the 299 patients not attaining pCR in the NeoSphere trial, 193 (65%) had successful GEP of residual disease (post‐treatment cohort), including 166 with paired specimens (Fig. 1). TRAR was computed starting from GEP data using 36 out of the 41 genes in the signature because 5 genes (*ARMET*, *C14orf173*, *C17orf37*, *C2orf48*, *WASH2P*) were not available in the Affymetrix platform. The missing genes were not in the core of the signature [11] and TRAR scores calculated with and without these genes in already analyzed datasets [11, 12] were significantly highly correlated (Fig. S1).

### 3.2 TRAR and treatment response in the overall cohort

**Fig. 2:**
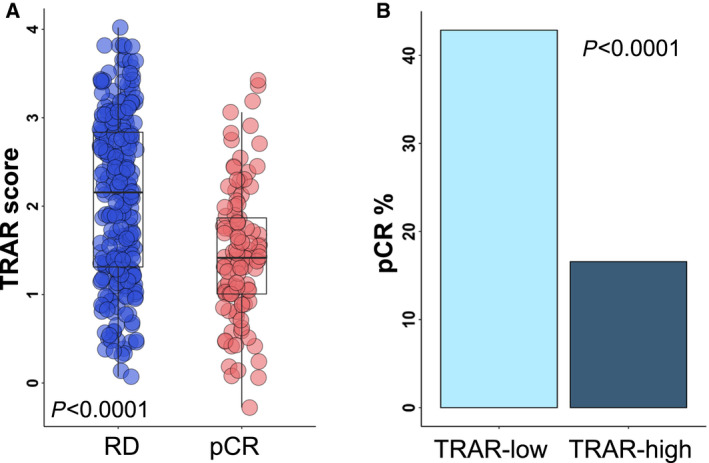
Predictive performance of TRAR. (A) Box‐plots of the distribution of TRAR score in patients with residual disease (RD) and pathological complete response (pCR) in the overall analyzed pre‐treatment cohort (*n* = 350). Shown are the 25th and the 75th percentiles of the distribution (box), the median (horizontal line), and the extreme values (whiskers). p‐value by Wilcoxon test. (B) Frequency of pCR in TRAR‐low and TRAR‐high subgroups. p‐value by chi‐square test.

In the pre‐treatment cohort, 104 (29.7%) patients achieved a pCR, specifically 27 (31%), 41 (45%), 14 (16%), and 22 (27%) following treatment with TH, THP, HP, and TP, respectively. As a continuous variable, TRAR was significantly lower in patients attaining a pCR overall (*P* < 0.0001) (Fig. 2A) and across the different treatment arms (Fig. S2). When TRAR was dichotomized, patients with TRAR‐low primary tumors (*n* = 175) resulted more likely to achieve a pCR as compared with those with TRAR‐high tumors (*n* = 175) (43% versus 17%, *P* < 0.0001, Fig. 2B). Univariate logistic regression analysis showed that the likelihood of attaining a pCR was 55% lower for each TRAR unit increment (OR: 0.45, 95% CI: 0.34‐0.60) (Table 1). In the multivariate analysis adjusted for relevant clinico–pathological characteristics including ER status, treatment arm, age and tumor type, TRAR and ER‐positive status remained each independently associated with lower pCR (OR 0.61 and 0.40, respectively), independently of other variables (Table 1).

**Table 1:** Association of TRAR and clinico‐pathological variables with pathological complete response (pCR): Univariate and multivariate logistic regression model. OR, odds ratio; CI, confidence interval; ER, estrogen receptor; T, taxanes; H, trastuzumab; P, pertuzumab; LABC, locally advanced breast cancer; IBC, inflammatory breast cancer.

All (*n* = 350)	Univariate		Multivariate*	
Biomarker	OR (95% CI)	*P*‐value	OR (95% CI)	*P*‐value
TRAR				
TRAR (continuous)	0.45 (0.34‐0.60)	3.57E‐08	0.61 (0.43‐0.88)	0.008
ER IHC				
ER IHC (pos vs neg)	0.24 (0.14‐0.40)	7.04E‐08	0.40 (0.20‐0.77)	0.006
Arm				
THP (vs TH)	1.79 (0.97‐3.30)	0.063	1.80 (0.93‐3.48)	0.084
HP (vs TH)	0.41 (0.20‐0.85)	0.016	0.41 (0.19‐0.87)	0.021
TP (vs TH)	0.83 (0.43‐1.62)	0.581	0.79 (0.38‐1.61)	0.512
Age				
Age (continuous)	0.98 (0.96‐1.01)	0.161	0.98 (0.96‐1.01)	0.188
Type				
LABC (vs OPERABLE)	1.28 (0.78‐2.09)	0.336	0.10 (0.58‐1.72)	0.993
IBC (vs OPERABLE)	0.94 (0.38‐2.35)	0.894	0.95 (0.34‐2.58)	0.909

ER‐positive (*n* = 161)	Univariate		Multivariate**	
Biomarker	OR (95% CI)	*P*‐value	OR (95% CI)	*P*‐value
TRAR				
TRAR (continuous)	0.33 (0.17‐0.63)	0.0008	0.40 (0.20‐0.79)	0.009
Arm				
THP (vs TH)	1.83 (0.59‐5.66)	0.292	1.56 (0.47‐5.17)	0.472
HP (vs TH)	0.26 (0.05‐1.38)	0.115	0.24 (0.04‐1.33)	0.102
TP (vs TH)	1.03 (0.30‐3.53)	0.960	0.75 (0.20‐2.83)	0.668
Age				
Age (continuous)	0.96 (0.92‐1.03)	0.070	0.97 (0.93‐1.02)	0.279
Type				
LABC (vs OPERABLE)	1.14 (0.43‐2.98)	0.797	0.99 (0.34‐2.87)	0.990
IBC (vs OPERABLE)	0.00 (0.00‐Inf)	0.988	0.00 (0.00‐Inf)	0.992

ER‐negative (*n* = 189)	Univariate		Multivariate**	
Biomarker	OR (95% CI)	*P*‐value	OR (95% CI)	*P*‐value
TRAR				
TRAR (continuous)	0.79 (0.52‐1.20)	0.259	0.79 (0.51‐1.21)	0.269
Arm				
THP (vs TH)	1.90 (0.86‐4.20)	0.114	1.84 (0.82‐4.11)	0.138
HP (vs TH)	0.45 (0.19‐1.08)	0.075	0.44 (0.18‐1.69)	0.070
TP (vs TH)	0.76 (0.33‐1.77)	0.526	0.74 (0.31‐1.73)	0.486
Age				
Age (continuous)	0.99 (0.96‐1.02)	0.515	0.99 (0.96‐1.02)	0.609
Type				
LABC (vs Operable)	1.06 (0.57‐1.93)	0.864	0.98 (0.52‐1.86)	0.959
IBC (vs Operable)	1.68 (0.53‐5.35)	0.379	1.78 (0.53‐5.98)	0.352

Multivariate analysis adjusted by (*) ER, treatment arm, age and type; (**) treatment arm, age and type.

### 3.3 TRAR and treatment response in ER‐positive and ER‐negative subgroups

**Fig. 3:**
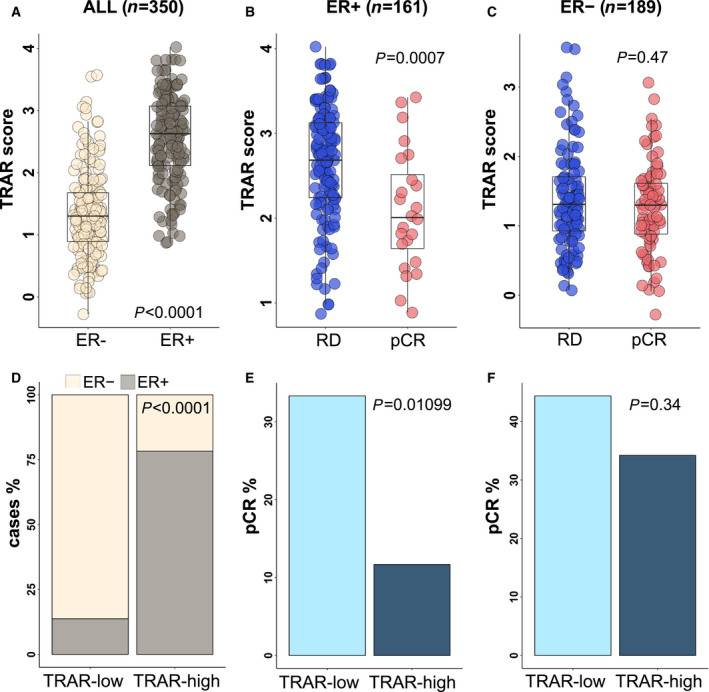
Predictive performance of TRAR according to tumor ER expression. (A) Box‐plots of the distribution of TRAR score in pre‐treatment patients (*n* = 350) with estrogen receptor‐positive (ER+) and estrogen receptor‐negative (ER−) tumors. *P*‐value by Wilcoxon test. (B‐C) Box‐plots of the distribution of TRAR score in patients with residual disease (RD) and pathological complete response (pCR) in the ER+ (*n* = 161, B) and ER− (*n* = 189, C) cohorts. *P*‐values by Wilcoxon test. (D) Frequency of ER status in TRAR‐low and TRAR‐high groups (*n* = 350). *P*‐value by chi‐square test. (E‐F) Frequency of pCR in patients with TRAR‐low and TRAR‐high and ER+ (*n* = 161, E) and ER− (*n* = 189, F) tumors. *P*‐values by chi‐square test.

TRAR was significantly associated with ER both as a continuous and as a dichotomized variable (Fig. 3). TRAR scores were significantly higher in ER‐positive (*n* = 161) as compared to ER‐negative (*n* = 189) cases (*P* < 0.0001) (Fig. 3A and D). TRAR scores were significantly associated with pCR in ER‐positive both as continuous (*P* = 0.0007) (Fig. 3B) and dichotomous variable (*P* = 0.01099) (Fig. 3E), but not in ER‐negative cases (Fig. 3C and F) (test for interaction, *P* = 0.0280 and *P* = 0.1547, respectively). In ER‐positive cases, TRAR was associated with lower pCR (OR: 0.33, 95% CI: 0.17–0.63) in univariate analysis, (Table 1). In multivariate analysis, a significant association with lower pCR was found only for TRAR (OR: 0.40, 95% CI: 0.20–0.79, Table 1). In ER‐negative tumors, no variables were significantly associated with pCR (Table 1). Similar results were obtained with TRAR dichotomous variable: TRAR‐high was associated with lower pCR only in ER‐positive cases both in univariate (OR: 0.26, 95% CI: 0.10–0.72) and in multivariate analyses (OR: 0.33, 95% CI: 0.11–0.96, Table S2).

### 3.4 Changes of TRAR score in patients not achieving pCR

**Fig. 4:**
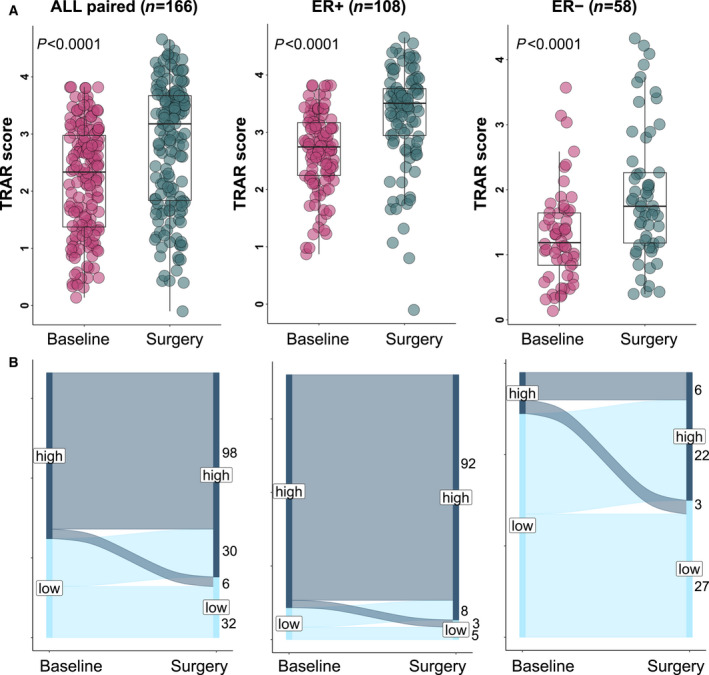
TRAR modulation by treatment in patients with residual disease at surgery. (A) Box‐plots of the distribution of TRAR score in pre‐treatment biopsies (baseline) and at surgery in the overall cohort (*n* = 166), in ER+ (*n* = 108) and in ER− (*n* = 58) subgroups. *P*‐values by Wilcoxon test. (B) Alluvial diagram of the change in TRAR classification between basal biopsies (Baseline) and samples at surgery in the overall cohort (*n* = 166), in ER+ (*n* = 108) and in ER− (*n* = 58) subgroups.

The post‐treatment cohort (*n* = 193) differed from the pre‐treatment cohort (*n* = 350), with fewer TRAR‐low cases (24% vs 50%, *P* = 0.0005), as expected given high pCR rates among this subgroup, even among ER‐positive cases (8% vs 15%, *P* = 0.09). Comparison of matched pre‐ and post‐treatment tumors (*n* = 166) demonstrated a significant increase in TRAR scores in post‐treatment cohort overall, according to ER status (Fig. 4A) and treatment arms (Fig. S3). Specifically, a TRAR switch from low to high status occurred in 30/62 (48%) cases, while the opposite in 6/104 (6%) cases (*P* < 0.0001); among ER‐positive cases 8/13 (62%) turned to TRAR‐high, and 3/95 (3%) to TRAR‐low (*P* = 0.0005), while the corresponding rates were 22/49 (45%), and 3/9 (33%) in ER‐negative cases (*n* = 58) (Fig. 4B).

**Fig. 5:**
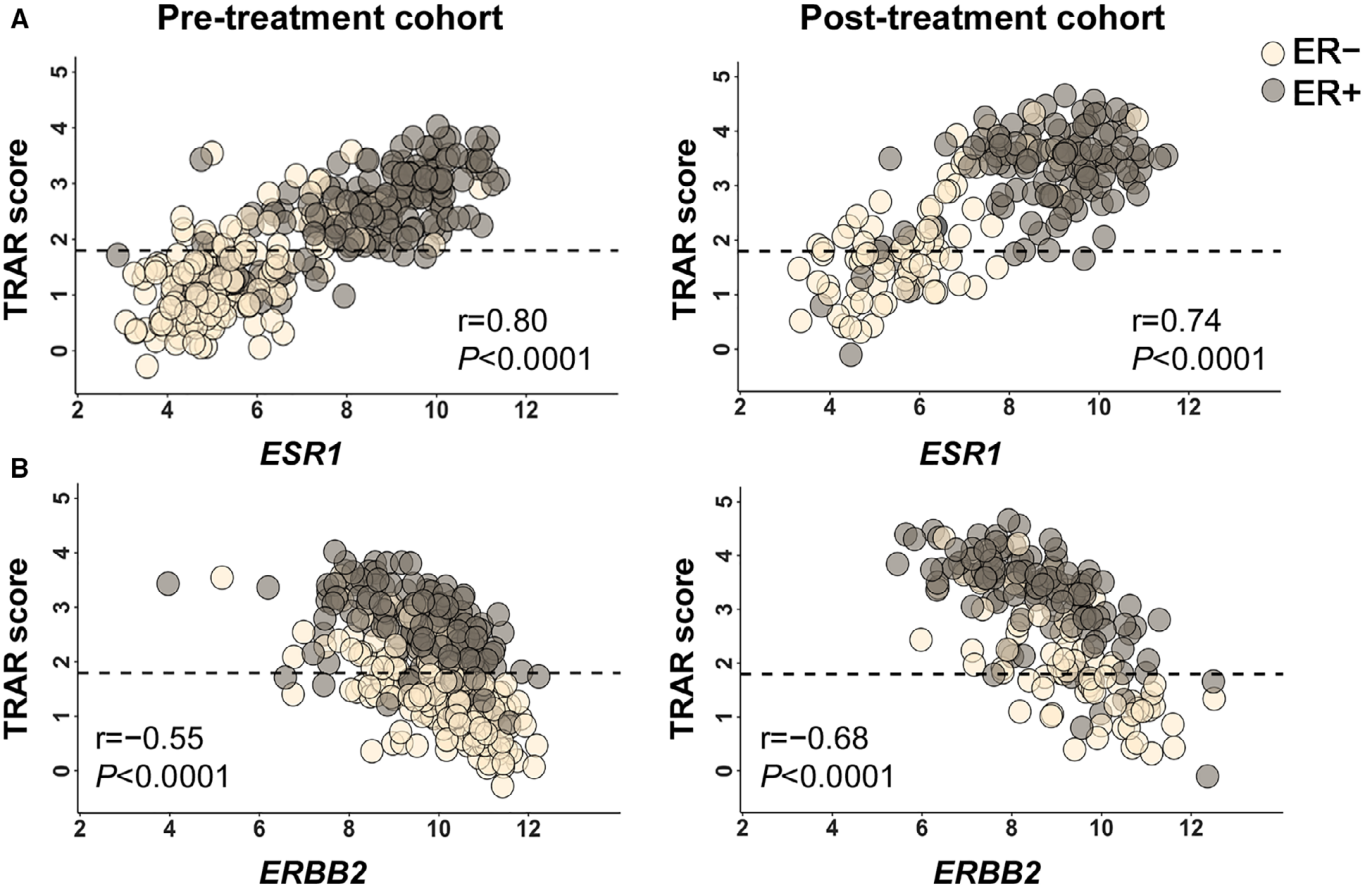
Correlation between TRAR score, *ESR1* and *ERBB2* genes. (A‐B) Pearson correlation analysis between TRAR score and *ESR1* (A) and *ERBB2* (B) in pre‐treatment (*n* = 350) and post‐treatment (*n* = 193) cohorts according to ER status. Horizontal dot lines separate TRAR‐low from TRAR‐high patients. *r*, Pearson correlation coefficients and related *P*‐values are shown.

Of note, TRAR correlated with the expression levels of *ESR1* and *ERBB2* in both the pre‐treatment (*r* = 0.80, *P* < 0.0001 and *r* = −0.55, *P* < 0.0001, respectively) and post‐treatment (*r* = 0.74, *P* < 0.0001 and *r* = −0.68, *P* < 0.0001, respectively) cohorts. Nevertheless, while unresponsive ER‐positive cases classified as TRAR‐low at surgery tended to maintain the same low *ESR1* and high *ERBB2* expression levels of their pre‐treatment counterparts, those classified as TRAR‐high at surgery were more likely to show reduced levels of *ERBB2* expression as compared to pre‐treatment counterparts (Fig. 5). TRAR–low tumors expressed significantly higher level of proliferation genes than TRAR‐high tumors (Fig. S4). Notably, while proliferation metagene levels were significantly decreased in TRAR‐high tumors at surgery compared to baseline (*P* < 0.0001), the opposite was observed in TRAR‐low cases with a significant increase in proliferation level at surgery (*P* = 0.0096) (Fig. S4).

### 3.5 TRAR and DEFS

At a median follow‐up of 61 months (range 60–63), no significant difference in terms of DEFS was found according to TRAR subgroups (Fig. S5A). Nevertheless, among ER‐positive cases, patients with TRAR‐high tumors at baseline in pre‐treatment cohort, despite achieving the lowest pCR rate (12%), tended to have a longer DEFS as compared to those with TRAR‐low tumors (HR: 0.41, 95% CI: 0.16–1.05, *P* = 0.06). The prognosis of patients with TRAR‐low tumors was especially dismal when baseline TRAR‐low status remained unaltered by neoadjuvant treatment (Fig. S5B), suggesting a potential intrinsic resistance to the HER2 targeted therapy. To further examine the relationship between TRAR and prognosis, we tested the outcome according to pCR (Fig. S5C). Remarkably, there was no difference in terms of DEFS among patients achieving a pCR, whereas in the absence of pCR, a trend toward worse outcome (*P* = 0.1259) was seen among TRAR‐low over TRAR‐high patients.

## 4 Discussion

Our findings confirm the predictive value of the TRAR classifier already identified in small retrospective studies [11] and in the large phase III NeoALTTO trial [12]. TRAR significantly associated with pCR independently of treatment arm, both as continuous and as dichotomized variable. Notably, the predictive value of TRAR showed a significant interaction with ER status. To our knowledge, this is the first report to suggest that a parsimonious gene assay might help to identify a subset of patients with nonmetastatic HER2‐ and ER‐positive disease that can benefit the most from HER2‐targeted based neoadjuvant chemotherapy.

TRAR score was significantly different in the ER‐positive and ER‐negative subgroups, supporting the described differences in gene expression pattern of HER2‐positive breast cancer according to ER expression [15, 16]. These differences may explain the lower pCR rates reported in ER‐positive as compared to ER‐negative cases in the NeoSphere and other trials, including NeoALTTO [17] and GEPARSIXTO [18]. Notably, TRAR is likely to identify a group of HER2‐positive and ER‐positive tumors that are not exquisitely driven by ER signaling and thus benefit the most from HER2 targeting therapies. This could be explained by the ability of TRAR to mirror not only ER expression/function through the expression of *ESR1* and related genes but also HER2 and related genes also recapitulating their interplay, which is relevant in response determination [19, 20].

It is important to note that we could not exclude a benefit of applying TRAR to patients with ER‐negative tumors, as we recently reported in other dataset [12]. Indeed, contrary to other studies [21, 22, 23, 24], in the NeoSphere trial *ERBB2*, which is a core gene of TRAR score, was not significantly associated with pCR [25], and the PAM50 molecular classifier provided no predictive information overall [26]. Moreover, TRAR was previously tested in tumor biopsy of patients treated with chemotherapy in combination with trastuzumab [11] or other type of dual blockade, that is, trastuzumab plus lapatinib [12]. Therefore, the predictive value of TRAR only in ER‐positive tumors of NeoSphere study might be dependent on the anti‐HER2 drug used and limited to dual HER2‐blocking antibodies. Hence, prospective evaluation of the predictive findings of our study in a randomized trial is warranted, as no similar cohort are currently available for external validation.

Intriguingly, a trend toward lower DEFS was found for TRAR‐low cases, though the study was underpowered for survival. The paradox of higher sensitivity to neoadjuvant HER2‐targeted therapy and poor prognosis in the TRAR‐low subgroup could be explained by the high relapse among those with residual disease, as already observed for HER2‐enriched cases in CALGB 40601 and NeoSphere trials [21, 26]. Hence, it may be easier to achieve pCR in TRAR‐low tumors, especially in ER‐positive cases, but if pCR does not occur, then patients are more likely to relapse early. This finding is in line with the baseline aggressive features of TRAR‐low tumors, that is, high HER2 oncogene dependence, low ER activity, and high proliferation [11] and their poor prognosis if untreated [11].

From the clinical point of view, this finding leads to three major considerations. First, TRAR at surgery may reflect the importance of adjuvant endocrine therapy, which probably exerts its benefit in patients with TRAR‐high remnants, which increased luminal features after neoadjuvant therapy, rather than TRAR‐low remnants that being intrinsically resistant to anti‐HER2 therapies maintain low *ESR1* expression and increase proliferation. Interestingly, higher pCR rate but also higher risk of relapse despite chemoendocrine treatments were predicted among ER‐positive/HER2‐negative patients by other genomic classifiers related to proliferation and ER activity [14, 27, 28], supporting a role for these features in explaining TRAR‐low poor prognosis. Next, genomic characterization of surgical specimens could help to identify actionable targets and to foster drug development for residual tumors after HER2‐targeted (neoadjuvant) therapy. In this sense, the HER2‐targeted therapy‐induced luminal phenotype has been already associated to increase sensitivity to CDK4/6 inhibition [29, 30]. Finally, if TRAR prognostic value will be validated, TRAR could be developed as a tool to aid salvage adjuvant treatment, which has already proven to ameliorate patient prognosis at least in a portion of cases not achieving pCR [31]. This adjuvant treatment escalation could be especially relevant for patients with TRAR‐low tumors at surgery.

Some caveats of our study require special consideration. The different platform did not allow us to test the validity of previous generated TRAR cutoff and the use of the median cutoff could have reduced its predictive value. Moreover, the small sample size of treatment arms, and the lack of a validation series did not allow to test whether the interaction between TRAR and ER is limited to the dual HER2‐blocking antibodies.

## 5 Conclusion

In conclusion, we independently confirm in NeoSphere trial, the clinical validity of TRAR in predicting pCR to anti‐HER2‐based chemotherapy beyond that provided by standard pathologic markers. TRAR represents a promising tool to stratify ER‐positive/HER2‐positive patients by likelihood of response to anti‐HER2‐based neoadjuvant therapy and to contribute to define treatment escalation and de‐escalation strategies in this setting. The clinical utility of this genomic test in predicting also long‐term benefit (i.e., DEFS and overall survival) warrants further investigation.

## Conflict of interest

GB: Roche, MSD, Pfizer, AstraZeneca, Lilly, Novartis, Neopharm, Amgen, Chugai, Sanofi, Daiichi Sankyo, EISAI, Genomic Health (consultant/advisory board member); SDC: Novartis Pharma and Pierre Fabre (speaking fees); TP: Roche (honorarium fee, grants, travel grant); GVB: Novartis and Eli Lilly (consultant/advisory board member); MCL: Roche and Pfizer (advisory board member, travel grant); BB: Roche, MSD, Novartis (consultant/advisory board member), Roche, Novartis, MSD, Pfizer, Pierfabre (speakers’ bureau), Pfizer (travel grant); DYO: AstraZeneca, Novartis, Genentech/Roche, Merck Serono, Bayer, Taiho, ASLAN, Halozyme, Zymeworks, BMS/Celgene, BeiGene, Basilea, Turning Point (consultant/advisory board member), AstraZeneca, Novartis, Array, Eli Lilly, Servier, BeiGene, MSD, Handok (research grant); LG: ADC Therapeutics, Amgen, AstraZeneca, Biomedical Insights Inc, Celgene, Eli Lilly, G1 Therapeutics, Genentech, GENENTA, Genomic Health, Menarini Ricerche, METIS Precision Medicine, Merck Sharp & Dohme, Novartis, Oncolytics Biotech, Odonate Therapeutics, Onkaido Therapeutics, Revolution Medicines, Roche, Pfizer, Taiho Pharmaceutical, Hexal Sandoz, Seattle Genetics, selected programs of Forty Seven (CD47), Synthon, Synaffix, Zymeworks and Sanofi‐Aventis (consultant/advisory board member); TT, YHI, GB, MD, LDC, LMT,VS, GV, JdlHR, BP, PV and ET declare no competing interest.

## Author contributions

TT, GB, SDC, LG, and ET conceived the work; TT, GB, BG, MD, LDC, and GV performed analyses; TT, GB, SDC, LG, and ET interpreted the results; TP, YHI, GVB, LMT, MCL, BB, VS, JdlHR, DYO, BP, and LG provide resources; GB and PV managed data; TT, SDC, and ET wrote the manuscript; GB, TP, YI, GVB, BG, MD, LDC, LMT, MCL, BB, VS, GV, JdlHR, DYO, BP, and PG critically revised the manuscript; all authors approved the final version of the manuscript.

### Peer review

The peer review history for this article is available at https://publons.com/publon/10.1002/1878‐0261.13141.

## Supporting information


**Fig. S1**. TRAR scores according to signature composition.
**Fig. S2**. Predictive performance of TRAR according to treatment arm.
**Fig. S3**. TRAR modulation according to treatment arm.
**Fig. S4**. Proliferation metagene according to TRAR.
**Fig. S5**. Prognostic performance of TRAR.

**Table S1**. Expression of genes used to calculate TRAR score and proliferation metagene.

**Table S2**. Association of TRAR and clinico‐pathological variables with pathological complete response (pCR): Univariate and multivariate logistic regression model.

## Data Availability

Gene expression data used to calculate TRAR score and proliferation metagene are available in the supplementary material of this article; clinical data are available upon reasonable request to Fondazione Michelangelo.
