# Supplementary material for: The TRAR gene classifier to predict response to neoadjuvant therapy in HER2‐positive and ER‐positive breast cancer patients: an explorative analysis from the NeoSphere trial
Source: Mol Oncol. 2021 Dec 17;16(12):2355–66. doi: 10.1002/1878-0261.13141 (PMC9208076; doi:10.1002/1878-0261.13141)
Supplement: Supplementary file 1 — Fig. S1. TRAR scores according to signature composition. Fig. S2. Predictive performance of TRAR according to treatment arm. Fig. S3. TRAR modulation according to treatment arm. Fig. S4. Proliferation metagene according to TRAR. Fig. S5. Prognostic performance of TRAR. [file MOL2-16-2355-s002.pdf]

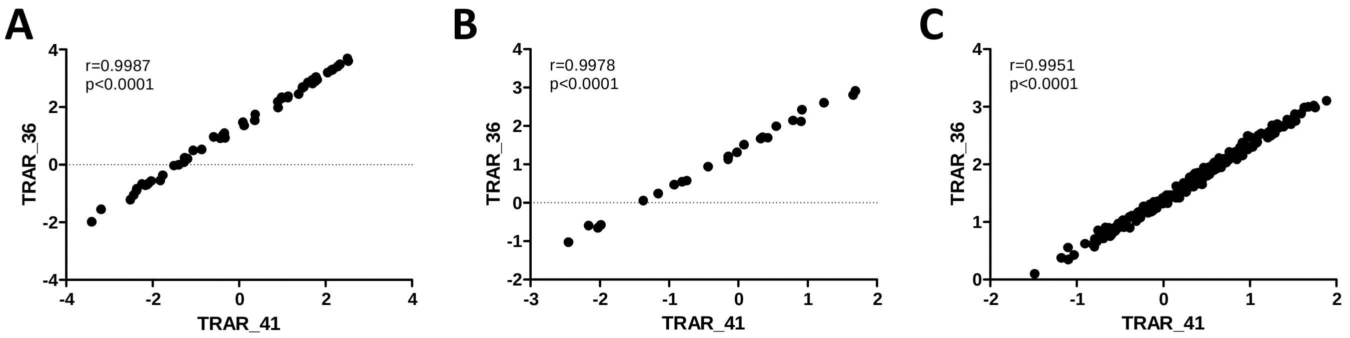

Supplementary Figure 1. TRAR scores according to signature composition. Correlation between TRAR_41 (computed using all genes of the signature) and TRAR_36 (computed using 36 genes of the signature as done in the NeoSphere cohort) in GHEA (A, n=54), TRUP (B, n=24) and NeoALTTO (C, n=226) datasets. r, Pearson correlation coefficients and related p-values are shown.

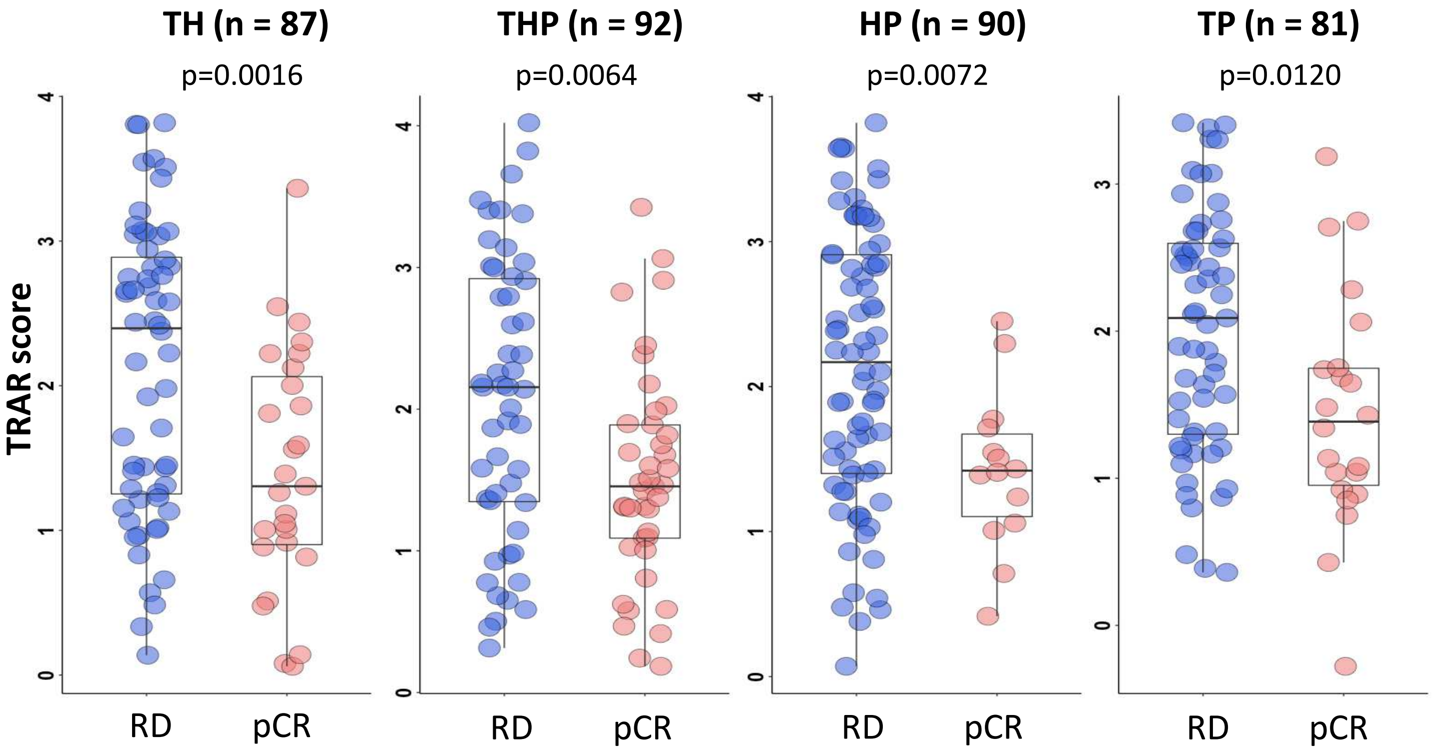

Supplementary Figure 2. Predictive performance of TRAR according to treatment arm. Box-plots of the distribution of TRAR score in patients with residual disease (RD) and pathological complete response (pCR) in each Neosphere treatment arm. p-values by Wilcoxon test. T, taxane; H, trastuzumab; P, pertuzumab.

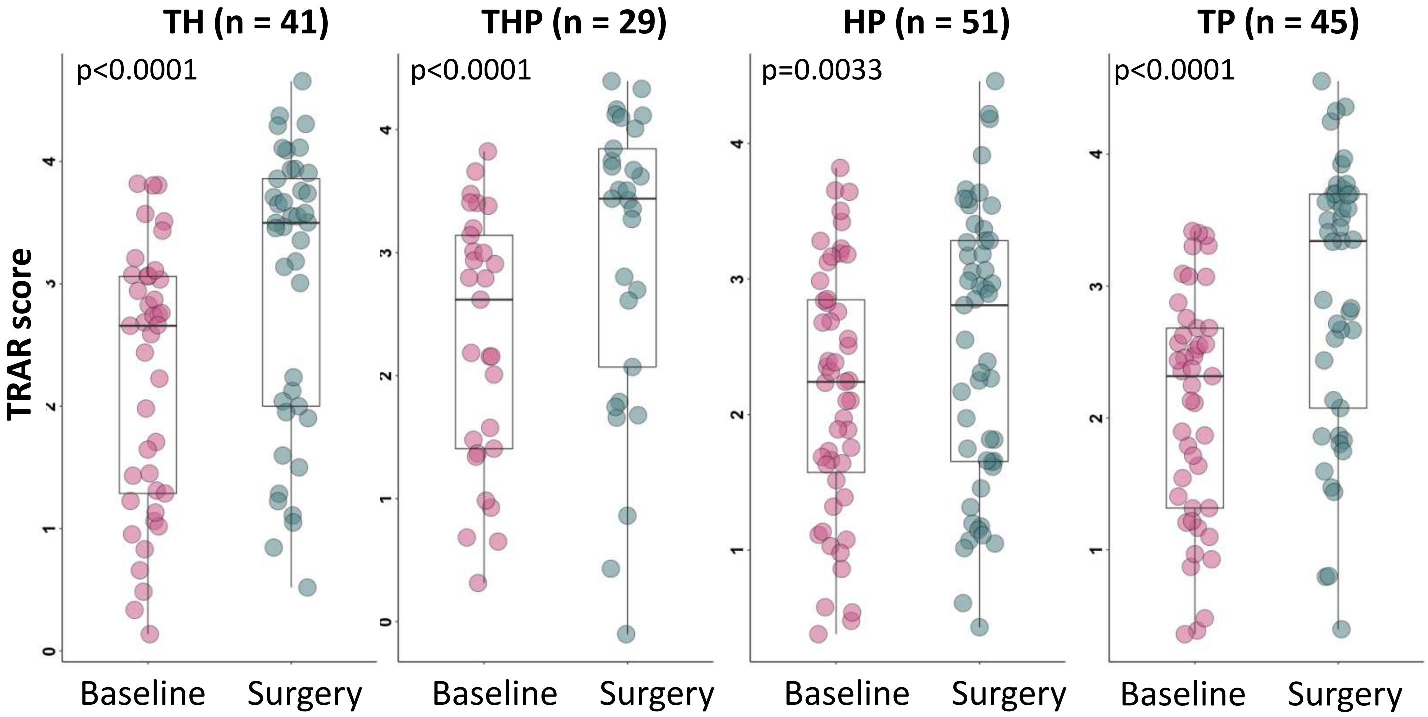

Supplementary Figure 3. TRAR modulation according to treatment arm. Box-plots of the distribution of TRAR score as evaluated in pre-treatment biopsies (Baseline) and at surgery of patients enrolled in each Neosphere treatment arm. p-values by Wilcoxon test. T: taxane, H: trastuzumab, P: pertuzumab.

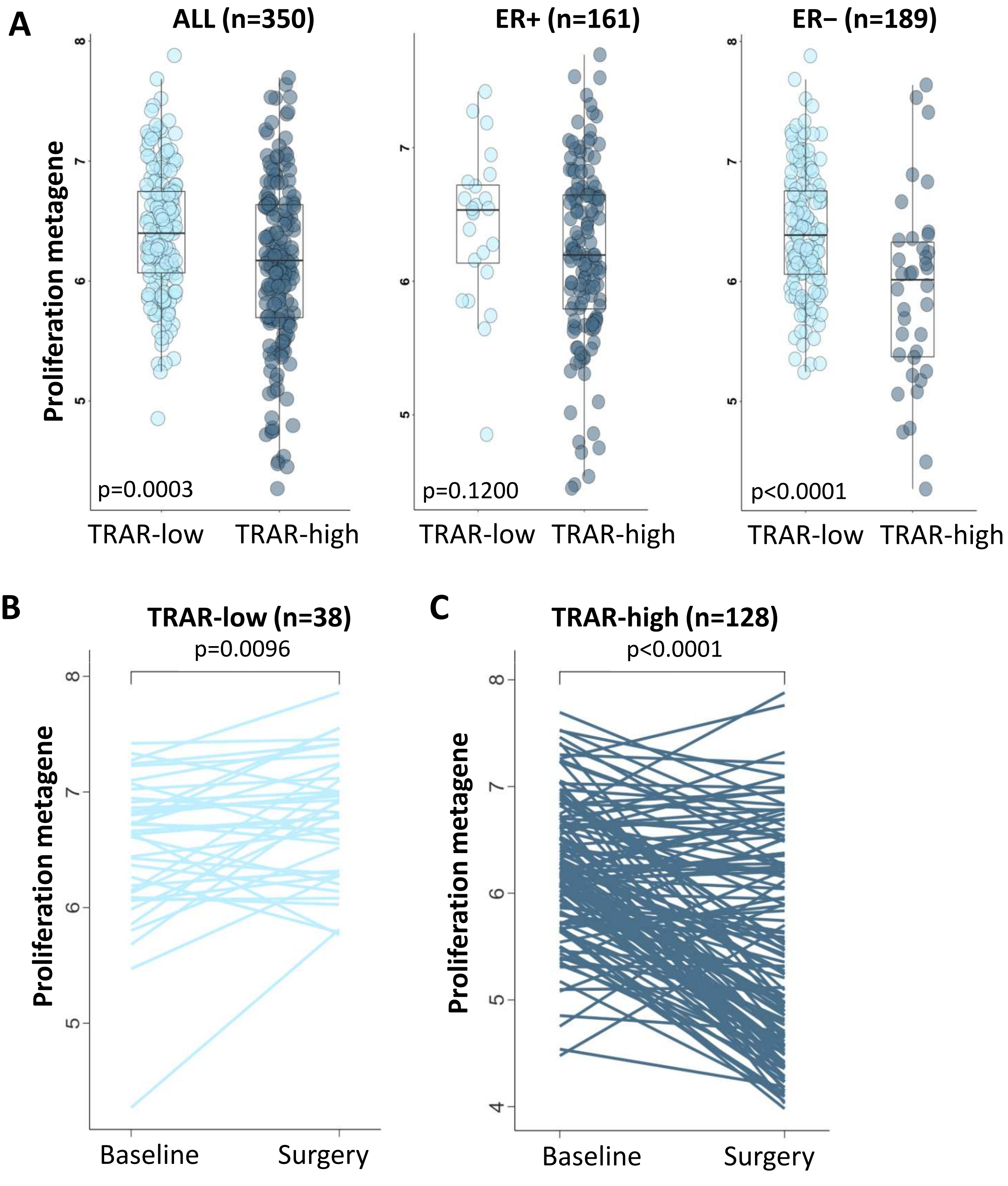

Supplementary Figure 4. Proliferation metagene according to TRAR. Box-plots of the distribution of proliferation metagene expression in TRAR-low and TRAR-high tumors at baseline in the overall cohort (n=350), in ER+ (n=161) and ER− (n=189) subgroups. p-values by Wilcoxon test. B-C) Spaghetti plots of the change in proliferation metagene between basal biopsies (Baseline) and samples at surgery in TRAR-low (n=38, B) and TRAR-high (n=128, C) tumors. p-values by paired Wilcoxon test.

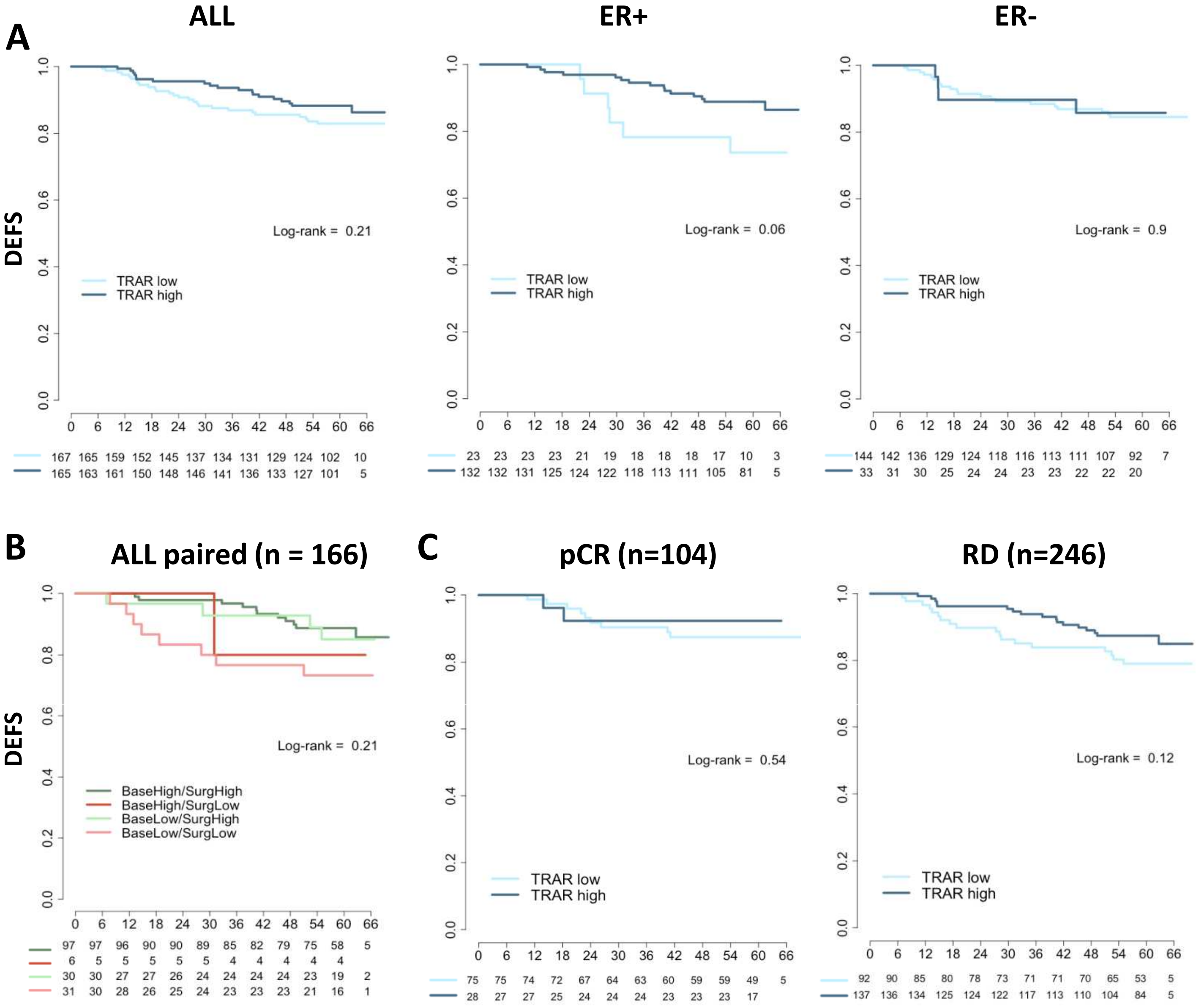

Supplementary Figure 5. Prognostic performance of TRAR. A) Kaplan-Meier estimates of distant event-free survival (DEFS) according to TRAR in the pre-treatment cohorts. B) Kaplan-Meier estimates of DEFS in 166 matched cases according to modulation of TRAR. C) Kaplan-Meier estimates of DEFS according to TRAR in patients of the pre-treatment cohort who achieved pCR (pCR) or not (RD). p-values by Log-Rank test.
