# Supplementary material for: The TRAR gene classifier to predict response to neoadjuvant therapy in HER2‐positive and ER‐positive breast cancer patients: an explorative analysis from the NeoSphere trial
Source: Mol Oncol. 2021 Dec 17;16(12):2355–66. doi: 10.1002/1878-0261.13141 (PMC9208076; doi:10.1002/1878-0261.13141)
Supplement: Supplementary file 3 — Table S2. Association of TRAR and clinico‐pathological variables with pathological complete response (pCR): Univariate and multivariate logistic regression model. [file MOL2-16-2355-s003.docx]

**Supplementary Table 2. Association of TRAR and clinico-pathological variables with pathological complete response (pCR): Univariate and multivariate logistic regression model**

| **ALL (n=350)** | |  | **UNIVARIATE** | |  | **MULTIVARIATE*** | | | | | | | | | | |  |  |
| --- | --- | --- | --- | --- | --- | --- | --- | --- | --- | --- | --- | --- | --- | --- | --- | --- | --- | --- |
| Biomarker | |  | OR (95% CI) | p-value |  | OR (95% CI) p-value | | | | | | | | | | |  |  |
| **TRAR** | |  |  |  |  |  | | | |  | | | | | | |  |  |
| TRAR-high (vs TRAR-low) | |  | 0.27 (0.16-0.44) | 1.7E-07 |  | 0.45 (0.24-0.85) | | | | 0.014 | | | | | | |  |  |
| **ER IHC** | |  |  |  |  |  | | | |  | | | | | | |  |  |
| ER IHC (pos vs neg) | |  | 0.24 (0.14-0.40) | 7.04E-08 |  | 0.37 (0.19-0.72) | | | | 0.003 | | | | | | |  |  |
| **Arm** | |  |  |  |  |  | | | |  | | | | | | |  |  |
| THP (vs TH) | |  | 1.79 (0.97-3.30) | 0.063 |  | 1.79 (0.92-3.46) | | | | 0.085 | | | | | | |  |  |
| HP (vs TH) | |  | 0.41 (0.20-0.85) | 0.016 |  | 0.39 (0.18-0.84) | | | | 0.016 | | | | | | |  |  |
| TP (vs TH) | |  | 0.83 (0.43-1.62) | 0.581 |  | 0.77 (0.38-1.58) | | | | 0.473 | | | | | | |  |  |
| **Age** | |  |  |  |  |  | | | |  | | | | | | |  |  |
| Age (continuous) | |  | 0.98 (0.96-1.01) | 0.161 |  | 0.98 (0.96-1.01) | | | | 0.123 | | | | | | |  |  |
| **Type** | |  |  |  |  |  | | | |  | | | | | | |  |  |
| LABC (vs OPERABLE) | |  | 1.28 (0.78-2.09) | 0.336 |  | 1.04 (0.60-1.80) | | | | 0.881 | | | | | | |  |  |
| IBC (vs OPERABLE) | |  | 0.94 (0.38-2.35) | 0.894 |  | 0.95 (0.35-2.60) | | | | 0.922 | | | | | | |  |  |
|  |  | | | | | | | |  | | | | | | | |  |  |
| **ER-positive (n=161)** | |  | **UNIVARIATE** | |  | | | **MULTIVARIATE**** | | | | | | | | |  |  |
| Biomarker | |  | OR (95% CI) | p-value |  | | | OR (95% CI) | | | | | | p-value | | |  |  |
| **TRAR** | |  |  |  |  | | |  | | | | |  |  |  |  |  |  |
| TRAR-high (vs TRAR-low) | | | 0.26 (0.10-0.72) | 0.0089 |  | | | 0.33 (0.11-0.96) | | | | | 0.041 | | |  |  |  |
| **Arm** | |  |  |  |  | | |  | | | | |  | | |  |  |  |
| THP (vs TH) | |  | 1.83 (0.59-5.66) | 0.292 |  | | | 1.51 (0.46-4.93) | | | | | 0.493 | | |  |  |  |
| HP(vs TH) | |  | 0.26 (0.05-1.38) | 0.115 |  | | | 1.19 (0.03-1.08) | | | | | 0.061 | | |  |  |  |
| TP(vs TH) | |  | 1.03 (0.30-3.53) | 0.960 |  | | | 0.71 (0.19-2.66) | | | | | 0.610 | | |  |  |  |
| **Age** | |  |  |  |  | | |  | | | | |  | | |  |  |  |
| Age (continuous) | |  | 0.96 (0.92-1.03) | 0.070 |  | | | 0.97 (0.92-1.01) | | | | | 0.147 | | |  |  |  |
| **Type** | |  |  |  |  | | |  | | | | |  | | |  |  |  |
| LABC (vs OPERABLE) | |  | 1.14 (0.43-2.98) | 0.797 |  | | | 1.11 (0.39-3.17) | | | | | 0.844 | | |  |  |  |
| IBC (vs OPERABLE) | |  | 0.00 (0.00-Inf) | 0.988 |  | | | 0.00 (0.00-Inf) | | | | | 0.988 | | |  |  |  |
|  |  | | | | | | | | | | | | | | | |  |  |
| **ER-negative (n=189)** | |  | **UNIVARIATE** | |  | | **MULTIVARIATE***** | | | | | | | | | | | |
| Biomarker | |  | OR (95% CI) | p-value |  | | | OR (95% CI) | | | | p-value | | | | | |  |
| **TRAR** | |  |  |  |  | | |  | | |  | | | |  |  |  |  |
| TRAR-high (vs TRAR-low) | | | 0.65 (0.31-1.37) | 0.259 |  | | | 0.60 (0.27-1.31) | | | 0.199 | | | |  |  |  |  |
| **Arm** | |  |  |  |  | | |  | | |  | | | |  |  |  |  |
| THP (vs TH) | |  | 1.90 (0.86-4.20) | 0.114 |  | | | 1.85 (0.83-4.14) | | | 0.133 | | | |  |  |  |  |
| HP(vs TH) | |  | 0.45 (0.19-1.08) | 0.075 |  | | | 0.44 (0.18-1.06) | | | 0.066 | | | |  |  |  |  |
| TP(vs TH) | |  | 0.76 (0.33-1.77) | 0.526 |  | | | 0.73 (0.31-1.72) | | | 0.471 | | | |  |  |  |  |
| **Age** | |  |  |  |  | | |  | | |  | | | |  |  |  |  |
| Age (continuous) | |  | 0.99 (0.96-1.02) | 0.515 |  | | | 0.99 (0.96-1.02) | | | 0.535 | | | |  |  |  |  |
| **Type** | |  |  |  |  | | |  | | |  | | | |  |  |  |  |
| LABC (vs OPERABLE) | |  | 1.06 (0.57-1.93) | 0.864 |  | | | 1.02 (0.54-1.93) | | | 0.959 | | | |  |  |  |  |
| IBC (vs OPERABLE) | |  | 1.68 (0.53-5.35) | 0.379 |  | | | 1.79 (0.53-6.02) | | | 0.348 | | | |  |  |  |  |

OR, odds ratio; CI, confidence interval; ER, estrogen receptor; T, taxanes; H, trastuzumab; P, pertuzumab; LABC, locally advanced breast cancer; IBC, inflammatory breast cancer.

Multivariate analysis adjusted by (*) ER, treatment arm, age and type; (**)treatment arm, age and type; (***) treatment arm, age and type.
